# Supplementary material for: Dissection of a Krox20 positive feedback loop driving cell fate choices in hindbrain patterning
Source: Mol Syst Biol. 2013 Sep 24;9:690. doi: 10.1038/msb.2013.46 (PMC3792346; doi:10.1038/msb.2013.46)
Supplement: Supplementary Information [file msb201346-s1.pdf]

SUPPLEMENTARY INFORMATION

Dissection of a Krox20 feedback loop driving cell fate choices in hindbrain patterning

Yassine Xavier Bouchoucha, Jürgen Reingruber, Charlotte Labalette, Michel Adam Wassef, Elodie Thierion, Carole Desmarquet-Trin Dinh, David Holcman, Pascale Gilardi-Hebenstreit and Patrick Charnay.

Supplementary inventory

The supplementary information contains a supplementary text that provides computational details on the stochastic model of Krox20 autoregulation, ten supplementary figures and their legends, two supplementary tables and their legends and references for supplementary information.

1) Supplementary Text (ST)
This part is subdivided into three sections:
Section 1: presentation of the equations used to model Krox20 autoregulation
Section 2: details on the values used for the main parameters
Section 3: deterministic and stochastic analyses of the model

2) Supplementary Figures

3) Supplementary Tables

4) Supplementary References

Supplementary Text

This supplementary text (ST) contains the details of the stochastic model used to extract information from the experimental data. More importantly, it provides the framework for the stochastic gene activation analysis within the positive feedback loop. This ST is divided into three parts: first, we derive the equations for *Krox20* production. In the second part, we provide details on the values used for the main parameters. In the third part, we present a deterministic as well as a stochastic analysis of *Krox20* activation within the regulatory feedback loop.

1. Mathematical model for *Krox20* transcriptional regulation

1.1. General model description

Krox20 expression is initially zero. During a transient initiation phase that lasts for a time t_I, a Krox20-independent production mechanism allows the activation of the autoregulatory loop. After this initiation phase, Krox20 production relies solely on autoregulation (Fig. 4A). During initiation, in addition to autoregulation, *Krox20* mRNA is produced with a supplemental Poissonian rate Φ_I. Each mRNA molecule is degraded with a Poissonian rate Ψ and leads to the production of Krox20 proteins with an effective Poissonian production rate ϕ. Each Krox20 protein is degraded with a rate ψ and can bind to element A that has N_b =4 binding sites. We assume that the state of element A can be characterized by the number s=0, 1, 2, 3, 4 of bound Krox20 molecules. The autoregulatory production of *Krox20* mRNA occurs with Poissonian rates $\Phi_{A,s} = \Phi_A \xi_s$ that depend on the state s of element A, where Φ_A is the maximal production rate and ξ_s is a vector that describes the modulation due to the state of element A. Binding and unbinding of Krox20 to element A are described by state dependent binding and unbinding rates λ_s and μ_s.

The full system is characterized by three variables: the state s of element A, the number m of *Krox20* mRNA and the number n of unbound Krox20 proteins. The joint probability $p_s(m,n,t)$ to find element A in state s with m *Krox20* mRNA molecules and n free Krox20 proteins satisfies the Master equation (Schuss, 1980)

$$\begin{aligned}\frac{dp_s}{dt}(m,n,t) &= \Phi_s p_s(m-1,n,t) + (m+1)\Psi p_s(m+1,n,t) - (\Phi_s + m\Psi)p_s(m,n,t)\\ &+m\phi p_s(m,n-1,t) + (n+1)\psi p_s(m,n+1,t) - (m\phi + n\psi)p_s(m,n,t)\\ &+\mu_{s+1}p_{s+1}(m,n-1,t) + (n+1)\lambda_{s-1}p_{s-1}(m,n+1,t) - (\mu_s + n\lambda_s)p_s(m,n,t)\end{aligned} \quad (1)$$

where the total mRNA production rate is $\Phi_s(t) = \Phi_I \theta(t_I - t) + \Phi_{A,s}$ ($\theta(t)$ is the Heaviside function). The first line in eq. 1 describes the production and degradation of a *Krox20* mRNA, the second line the production and degradation of a Krox20 protein, and the last one is the binding and unbinding of a Krox20 protein to element A.
Eq. 1 describes Krox20 production due to a single allele in the nucleus. With two identical alleles one has to solve the Master equation for the joint probability

$p_{s_1,s_2}(m,n,t)$, where s_1 and s_2 denote the states of allele 1and allele 2. Because of the symmetry $p_{s_1,s_2}(m,n,t) = p_{s_2,s_1}(m,n,t)$, the marginal probabilities describing the time evolution of each allele are identical, and we have

$$p_s(m,n,t) = \sum_{s'=0}^{N_b} p_{s,s'}(m,n,t) = \sum_{s'=0}^{N_b} p_{s',s}(m,n,t). \tag{2}$$

Due to the high dimension of the state space, it is difficult to analyze $p_{s_1,s_2}(m,n,t)$ even numerically, and we will therefore study the probability $p_s(m,n,t)$ defined in eq. 3 (see below). The most significant difference between the one-allele and the two-allele situation is the mRNA production rate, twice as high with two alleles. In first approximation, we treat the case with two alleles as a single-allele system, but with a twice as large mRNA production rate. With this approximation, the marginal probability $p_s(m,n,t)$ in eq. 2 satisfies eq. 1, but with doubled production rates Φ_I and Φ_A.

To study the dynamics of Krox20 production, we shall make the following approximations: first, when the number of free Krox20 proteins is large compared to the number of Krox20 binding sites on element A ($n >> N_b$ with one allele and $n >> 2N_b$ with 2 alleles) we can neglect in eq. 1 the change in the number of free Krox20 proteins n due to binding and unbinding to element A, and write

$$\begin{aligned}\frac{dp_s}{dt}(m,n,t) &\approx \Phi_s p_s(m-1,n,t) + (m+1)\Psi p_s(m+1,n,t) - (\Phi_s + m\Psi)p_s(m,n,t)\\ &+m\phi p_s(m,n-1,t) + (n+1)\psi p_s(m,n+1,t) - (m\phi + n\psi)p_s(m,n,t)\\ &+\mu_{s+1}p_{s+1}(m,n,t) + n\lambda_{s-1}p_{s-1}(m,n,t) - (\mu_s + n\lambda_s)p_s(m,n,t)\end{aligned} \tag{3}$$

Second, we assume that binding and unbinding to element A is fast enough compared to the time scale where the number of Krox20 proteins changes significantly. In that case we approximate $p_s(m,n,t) \approx p(m,n,t)p_s(n)$, where $p_s(n)$ is the steady state probabilities to find element A in state s for a given number of Krox20 proteins n, and $p(m,n,t)$ is the probability to find m mRNA molecules and n proteins at time t. The steady state condition for binding and unbinding from eq. 3 gives $\mu_{s+1}p_{s+1}(n) = n\lambda_s p_s(n)$, leading to the steady state distribution

$$p_s(n) = \frac{\prod_{j=0}^{s-1} n\gamma_j}{\sum_{i=0}^{N_b}\prod_{j=0}^{i-1} n\gamma_j}, \tag{4}$$

where

$$\gamma_i = \frac{\lambda_i}{\mu_{i+1}}, \quad i = 0,\ldots N_b - 1. \tag{5}$$

Using the probabilities $p_s(n)$, we obtain the effective *Krox20* mRNA production rates

$$\Phi_A(n) = \sum_{s=0}^{N_b} \Phi_{A,s}p_s(n) = \Phi_A \sum_{s=0}^{N_b} \xi_s p_s(n). \tag{6}$$

Finally, using the total rate $\Phi(n,t)=\Phi_I\theta(t_I-t)+\Phi_A(n)$, we obtain for the joint probability $p(m,n,t)$ the approximated Master equation

$$
\begin{aligned}
\partial_t p(m,n,t) = \Phi(n,t)p(m-1,n,t)+(m+1)\Psi p(m+1,n,t)-(\Phi(n,t)+m\Psi)p(m,n,t) \\
+m\phi p(m,n-1,t)+(n+1)\psi\, p(m,n+1,t)-(m\phi+n\psi)p(m,n,t)
\end{aligned}
\quad (7)
$$

from which we can derive the marginal probabilities for *Krox20* mRNA molecules, Krox20 proteins and the state of element A are

$$
\begin{aligned}
p(m,t) &= \sum_{s=0}^{N_b}\sum_{n=0}^{\infty} p_s(m,n,t)=\sum_{n=0}^{\infty} p(m,n,t), \\
p(n,t) &= \sum_{s=0}^{N_b}\sum_{m=0}^{\infty} p_s(m,n,t)=\sum_{m=0}^{\infty} p(m,n,t), \\
p_s(t) &= \sum_{m=0}^{\infty}\sum_{n=0}^{\infty} p_s(m,n,t)=\sum_{n=0}^{\infty} p(n,t)p_s(n).
\end{aligned}
\quad (8)
$$

1.2. Mean number of Krox20 mRNAs and proteins due to initiation

To compute the numbers of Krox20 mRNAs and proteins produced due to initiation $\bar{m}_I$ and $\bar{n}_I$, we set $\phi_A=0$ in eq. 3 and switch off the autoregulation loop, and we obtain the mean field equations

$$
\begin{aligned}
\frac{d}{dt}\bar{m}_I &= \Phi_I-\bar{m}_I\Psi \\
\frac{d}{dt}\bar{n}_I &= \bar{m}_I\phi-\bar{n}_I\psi
\end{aligned}
\quad (9)
$$

After a direct integration, we get at the end of the initiation period (at time $t = t_I$) the solutions

$$
\begin{aligned}
\bar{m}_I(t_I) &= \frac{\Phi_I}{\Psi}\left(1-e^{-\Psi t_I}\right) \\
\bar{n}_I(t_I) &= \frac{\phi}{\psi}\frac{\Phi_I}{\Psi}\left(1-e^{-\psi t_I}\right)-\frac{\phi}{\psi-\Psi}\frac{\Phi_I}{\Psi}\left(e^{-\Psi t_I}-e^{-\psi t_I}\right) \\
&= \frac{\phi}{\psi}\bar{m}_I(t_I)\left(1-\frac{\psi}{\psi-\Psi}\frac{e^{-\Psi t_I}-e^{-\psi t_I}}{1-e^{-\psi t_I}}\right)
\end{aligned}
\quad (10)
$$

1.3. Numerical evaluation

Obtaining an analytic evaluation of eq. 1, 3 or 7, with the autoregulatory loop, mRNA molecules and proteins, is not at hand with current methods. Furthermore, because the final probability that a cell eventually expresses Krox20 depends on the transient initiation phase, we have to consider the time development of the system. This

analysis is again extremely challenging and not at hand. A time-dependent solution for a much simpler autoregulated system consisting of proteins and a single gene that switches between two states has recently been presented (Ramos *et al*, 2011). An asymptotic time dependent analysis of a self-regulating system with mRNA molecules and proteins, when the mRNA lifetime is much faster compared to the protein lifetime, was further investigated in (Bokes *et al*, 2012a). A steady state analysis with mRNA molecules and proteins only, without feedback, can be found in (Bokes *et al*, 2012b).

The parameter values that we found (see next section) are such that we could not further simplify the system of DNA, mRNA and Krox20. Thus, in order to study the exact time dependent probability distribution, we decided to evaluate eq. 1, 3 and 7 numerically. We truncated the number of mRNA and proteins and integrated the truncated system of equations using the EXPOKIT software package (Sidje, 1998). During the simulation we monitored and adapted the mRNA and protein truncation level to keep the probability loss less than 1%. To simulate the stochastic behavior of single cells, we used the Gillespie algorithm (Gillespie, 2005) to simulate the chemical reactions underlying eq. 1. We refer to these single cell traces as “molecular dynamics simulations”.

All simulations shown in the main text were obtained using eq. 1. Interestingly, even for small numbers of Krox20 proteins, we did not observe significant differences between simulations performed with eq. 1, eq. 3 or eq. 7, as revealed in Figures S4B and S6J. Indeed, significant activation of element A occurs only when the number of Krox20 proteins reaches a value around 30-40, which is large compared to the 8 binding sites. Thus, for a low number of Krox20 proteins, autoregulation has only a small impact, explaining the similar results of simulations performed with eq.1, 3 or 7.

2. Parameter estimations

Equation 1 depends on a large number of parameters summarized in Supplementary Table S1. We shall now estimate these parameters using the following strategy: whenever possible, we use experimental data. If such a direct parameterization was not feasible, we checked the published literature for reliable values. Finally, we determined the remaining parameters indirectly using our analysis or simulation results. We now provide details on these estimations.

2.1. Duration of Krox20 initiation t_I

The duration of initiation phase in the endogenous condition was determined using the experimental data from Figure 2K. The red curve corresponds to the amount of *Krox20* mRNA produced only by initiator elements. The duration of initiation was defined as the time along the abscissa between the origin and the peak of initiation, which leads to

$$t_I = 80\,min. \qquad (11)$$

2.2. Krox20 mRNA and protein degradation rates Ψ *and* ψ

Transgenic *Tg(hsp:mKrox20* $_{HA}$*)*embryos were heat-shocked for 10 minutes at $38\,^{\circ}C$ and the amounts of *mKrox20* mRNA and protein were measured every hour by RT-qPCR and semi-quantitative western-blotting respectively. An exponential fit of the resulting curves revealed mRNA and protein half-lives of $\Psi = 1/60\,min^{-1}$ and $\psi = 1/65\,min^{-1}$ (data not shown). We therefore used the values

$$\Psi = \frac{1}{60}\min^{-1} \quad \psi = \frac{1}{65}\min^{-1} \tag{12}$$

2.3. Synergy for mRNA production ξ_s

The mRNA production rates $\Phi_{A,s} = \Phi_A \xi_s$ (*s*=0,…,4) represent the concerted recruitment of the transcriptional machinery (PolII and co-enzymes) onto the *Krox20* promoter as a function of the number of Krox20 proteins s bound to element A. The vector ξ describes the modulation of the production rates due to the number of bound Krox20 proteins. To determine ξ_s, we used the relative activities of element A after mutations of single sites, as described in the Results section (see Figure 3C,D). Element A activity resulting from a single mutation was decreased to 23% in mean. Combined mutations of two or three sites essentially abolished element A activity. Therefore, in state 0, 1 and 2, the element A-dependent production of Krox20 is null; in state 3, the element A drives 23% of the full production that is reached in state 4. In our chick electroporation experiments, the activation of the *Tg(cA::gfp)* reporter gene could be considered as saturated since addition of exogenous Krox20 by co-electroporation did not increase the level of reporter gene in r3 and r5 (data not shown). Therefore, the amount of Krox20 proteins was not limiting in these experiments, suggesting that cooperative binding events did not interfere with the results. The synergy among Krox20 binding sites revealed by our experiment can thus be entirely assigned to the concerted recruitment of the transcriptional apparatus. To conclude, these results were implemented as

$$\xi_s = (0,0,0,0.23,1). \tag{13}$$

2.4. Krox20 mRNA production rate Φ_A*, when element A is fully active*

To determine the value of Φ_A, we first estimated the steady-state number of mRNA per cell in wild type conditions. Deep sequencing mouse hindbrain mRNA at E8.5 (Le Men et al., in preparation) provided us with relative values of mRNA levels of *Krox20* versus six ubiquitous genes (*Hdac1, Hmgb3, Wbp4, Pgm3, Riok3, Eif4e*). Absolute mRNA values for these ubiquitous genes in mammalian cells were extracted from a quantitative analysis by Schwanhäusser and collaborators (Schwanhäusser *et al*, 2011). A linear regression curve was drawn between the absolute and the relative numbers of mRNA and used to obtain the absolute value of *Krox20* mRNA per cell: we obtained 7. Since Krox20-positive cells represent approximately one third of the hindbrain, we estimated that the average number of *Krox20* mRNA per positive cell is 21. Taking this value for *Krox20* mRNA when the two copies of element A are fully active, the production rate due to a single allele is

$$\Phi_A = \frac{21}{2}\Psi \approx 0.18\,min^{-1}. \quad (14)$$

2.5. Initiation rate Φ_I

Our experiments showed that in the endogenous situation (as opposed to heat-shock experiments), when element A becomes fully activated, the maximal *Krox20* mRNA levels in r3 and r5 are doubled compared to the mutant case with initiation alone (see Figure 2K). In mutant conditions the average number of mRNA at the end of initiation is $\overline{m}_I(t_I) = \frac{2\Phi_I}{\Psi}\left(1 - e^{-\Psi t_I}\right)$ (see eq. 10), where $2\Phi_I$ accounts for two alleles. In the endogenous case the average number of *Krox20* mRNAs after a long time is $\overline{m}_A = \frac{2\Phi_A}{\Psi}$. Using the experimental result together with $t_I = 80\,min$ and $\Psi = \frac{1}{60}min^{-1}$ we obtained

$$\frac{\overline{m}_A}{\overline{m}_I(t_I)} = 2 \Rightarrow \Phi_I = 0.7\Phi_A \quad (15)$$

In simulations where the initiation is endogenous, we use the values $\Phi_A = 0.18min^{-1}$ and $\Phi_I = 0.7\Phi_A$. However, in heat-shock experiments, the initiation strength depends on the temperature, and to simulate such conditions, we varied Φ_I.

2.6. Krox20 protein translation rate ϕ

Krox20 translation rate was inferred from a recently published work by Schwanhäusser and colleagues who have used parallel quantification of mRNA and proteins at a genome scale in mouse fibroblasts to determine absolute cell copy numbers, the half-lifes and the synthesis rates of mRNA and proteins of over 5,000 genes (Schwanhäusser *et al*, 2011; 2013). Considering the 43 zinc-finger transcription factors of this study, we calculated that the median translation rate was 34 proteins/mRNA/hour. We therefore use in our simulations the translation rate

$$\phi = \frac{34}{60}min^{-1}. \quad (16)$$

2.7. Cooperativity coefficients γ_i

To determine the coefficients γ_i defined in eq. 5 we used the data collected in the electromobility-shift assays experiments (Figure 3B and S4). In these experiments, at steady state, the fraction of DNA fragments that contained at least one bound Krox20 protein is

$$r = \frac{dk_1 + dk_2 + dk_3 + dk_4}{d_0}. \quad (17)$$

where dk_s is the concentration of DNA fragments bound with i Krox20 molecules, and d_0 is the overall concentration of DNA fragments in the solution. To compute the fraction r, we used the following model for binding and unbinding of Krox20 proteins to element A in wild type condition with N_b=4 binding sites (see also

(Gunawardena, 2005) for a similar analysis of multisite protein phosphorylations):

$$\begin{aligned}
\frac{d}{dt}dk_1 &= \tilde{\lambda}_0 k dk_0 - \tilde{\mu}_1 dk_1 - (\tilde{\lambda}_0 k dk_1 - \tilde{\mu}_2 dk_2) \\
\frac{d}{dt}dk_2 &= \tilde{\lambda}_1 k dk_1 - \tilde{\mu}_2 dk_2 - (\tilde{\lambda}_2 k dk_2 - \tilde{\mu}_3 dk_3) \\
\frac{d}{dt}dk_3 &= \tilde{\lambda}_2 k dk_2 - \tilde{\mu}_3 dk_3 - (\tilde{\lambda}_3 k dk_3 - \tilde{\mu}_4 dk_4) \\
\frac{d}{dt}dk_4 &= \tilde{\lambda}_3 k dk_3 - \tilde{\mu}_4 dk_4 \\
k &= k_0 - \sum_{n=1}^{4} n dk_n, \quad dk_0 = d_0 - \sum_{n=1}^{4} dk_n
\end{aligned} \tag{18}$$

where $\tilde{\lambda}_s$ are the in vitro forward binding rates, $\tilde{\mu}_s$ are the in vitro unbinding rates, k is the concentration of free Krox20 proteins, and k_0 the overall concentration of Krox20 proteins. The affinities of all binding sites are the same and only the number of Krox20 proteins that are bound to element A is relevant. At steady state, eq. 18 reduces to a system of non-linear equations for the dk_i that cannot be solved analytically. However, the experimental results revealed that a significant fraction of bound DNA is only present when Krox20 is abundant ($k_0 >> d_0$), in which case we can neglect Krox20 depletion due to binding and use $k \approx k_0$. With this approximation, the steady state equations simplify to

$$dk_{i+1} = \hat{k}_0 \tilde{\gamma}_i dk_i, \quad i = 0,...,N_b - 1 \tag{19}$$

where $\hat{k}_0 = \frac{k_0}{d_0}$ is the number of proteins per DNA, and

$$\tilde{\gamma}_i = \frac{d_0 \tilde{\lambda}_i}{\tilde{\mu}_{i+1}}, \quad i = 0,...,N_b - 1 \tag{20}$$

By solving eq. 19 we obtain

$$\frac{dk_i}{d_0} = \frac{\prod_{j=0}^{i-1} \hat{k}_0 \tilde{\gamma}_j}{\sum_{k=0}^{N_b} \prod_{j=0}^{k-1} \hat{k}_0 \tilde{\gamma}_j}$$

and from this we finally find the ratio

$$r = 1 - \frac{dk_0}{d_0} = 1 - \frac{1}{\sum_{i=0}^{N_b} \prod_{j=0}^{i-1} \hat{k}_0 \tilde{\gamma}_j} \tag{21}$$

We used the experimental measurements of r as a function of $\hat{k}_0$ shown in Supplementary Figure S5A to fit the values for $\tilde{\gamma}_i$ using eq. 21 for various N_b. With data for N_b=2 we fitted $\tilde{\gamma}_0$ and $\tilde{\gamma}_1$, then we used these values to fit $\tilde{\gamma}_2$ with data for N_b=3, and finally we used the data for N_b=4 to fit $\tilde{\gamma}_3$. Using the fitting procedure from Matlab, we found

$$[\tilde{\gamma}] = \left[\frac{1}{378}, \frac{1}{4.3}, \frac{1}{14.1}, \frac{1}{3.3}\right] \tag{22}$$

There is no indication that binding and unbinding of Krox20 proteins to element A in vitro are significantly different compared to the endogenous situation in a cell, and we will therefore use the constants $\tilde{\gamma}_i$ to estimate the in vivo coefficient γ_i defined in eq. 5. However, since the time scale of the forward binding depends on the mean first passage time of a Krox20 protein to find element A (Schuss *et al*, 2007; Reingruber *et al*, 2009), which itself depends on the complex nuclear organization, this time scale could be different in vitro compared to in vivo. We therefore introduced an overall factor β that accounts for a change of the time scale in vitro compared to in vivo, such that

$$\gamma_i = \beta\tilde{\gamma}_i \tag{23}$$

We do not have experimental data nor values from the literature which could put additional constraints on the value of β, and we shall estimate β in supplementary section 3.2 using numerical simulations.

2.8. Krox20 binding and unbinding rates λ_i and μ_i

The simulations presented in the SI were obtained using equation 7 that depends on the steady state probabilities $p_s(n)$ given in eq. 4. To perform simulations of the full Master equation 2, or to perform molecular dynamics simulations with binding and unbinding events, we additionally specified the binding and unbinding rates λ_i and μ_i. From steady state experiments we obtained γ_i. To estimate λ_i and μ_i, we simplified the representation the binding and unbinding process and considered that forward binding rate λ_i does not depend on the state of element A, such that $\lambda_i \approx \lambda$. Thus, the cooperativity in γ_i results only from the unbinding rates (we used eq. 5)

$$\mu_i = \frac{\lambda}{\gamma_{i-1}} = \frac{\lambda}{\beta}\frac{1}{\tilde{\gamma}_{i-1}} = \frac{\mu}{\tilde{\gamma}_{i-1}}, \quad i = 1,\ldots,N_b \tag{24}$$

where we introduced the rate constant $\mu = \frac{\lambda}{\beta}$ such that $\beta = \frac{\lambda}{\mu}$.

In summary, to model binding and unbinding events we used the expressions

$$\begin{aligned} \lambda_i &= \lambda \\ \mu_i &= \frac{\lambda}{\beta}\frac{1}{\tilde{\gamma}_{i-1}} \end{aligned} \tag{25}$$

2.9. Summary of the parameters

Using our own experimental results or relying on the literature, we could estimate the values of all parameters except for the forward binding rate λ and the ratio $\beta = \frac{\lambda}{\mu}$. Assuming that binding and unbinding to element A is fast compared to the time required for a significant change in the number of Krox20 proteins, it is sufficient to know β to investigate *Krox20* expression. We determined β in supplementary section 3.2 using numerical simulations of eq. 7. To perform molecular dynamics simulations or to evaluate the eqs.1 or 3, we additionally used $\lambda = 1\text{min}^{-1}$. The exact value of λ is not important as long as $1/\lambda$ is small compared to the time scale at which the number of mRNA and proteins changes significantly (around 60 minutes).

3. Analysis of *Krox20* activation

We investigated how Krox20 initiation and autoregulation determine cell fate. Interestingly, after initiation, autoregulation takes over without any basal production rate, in which case the state with vanishing *Krox20* expression is the only steady state: Indeed, any *Krox20* expression will eventually vanish due to fluctuations that drive the system to the state with zero Krox20 proteins and mRNA molecules. However, for a strong autoregulation, the probability for such a drastic event to occur is very small and the state with a large Krox20 expression last for a sufficient long time for patterning to occur.

To obtain insight into the mechanisms governing *Krox20* expression, we will derive the deterministic dynamical system for the mean number of proteins underlying eq. 7, which we will analyze numerically.

3.1. Analysis of the deterministic dynamical system

For the following analysis, we introduce the scaled parameters (26)

and scaled variables $$m_0 = \frac{\Phi_A}{\Psi}, \quad n_0 = m_0 \frac{\phi}{\psi}, \quad \alpha = \beta n_0, \quad \varepsilon = \frac{\Psi}{\psi}, \quad \chi = \frac{\Phi_I}{\Phi_A}$$

$$\tau = \Psi t, \quad \hat{m} = \frac{m}{m_0}, \quad \hat{n} = \frac{n}{n_0} \tag{27}$$

The numbers m_0 and n_0 of Krox20 mRNA and protein correspond to the average values when element A is fully activated, in which case the scaled variables $\hat{m}$ and $\hat{n}$ are of order one. Starting from the expression for $\Phi_A(n)$ given in eq. 6, we define the auxiliary function

$$f(x) = \sum_{s=0}^{N_b} \xi_s \frac{\prod_{j=0}^{s-1} x\tilde{\gamma}_j}{\sum_{i=0}^{N_b} \prod_{j=0}^{i-1} x\tilde{\gamma}_j} \tag{28}$$

such that $\Phi_A(n) = \Phi_A f(\beta n)$, where we used $\gamma_j = \beta\tilde{\gamma}_j$. The function *f(βn)* contains the synergy in *Krox20* mRNA production (the parameters ξ_s) and the cooperativity in binding and unbinding of Krox20 proteins to element A (the ratios γ_j). In Supplementary Figure S5B, we plotted two examples of $f(\beta n)$ for $\beta = 1$ and $\beta = 0.21$. $f(\beta n)$ represents the normalized production of element A (ranging from 0 to 1) as a function of the total number of Krox20 proteins.

We first approximate eq. 7 at first order and we neglect the initiation ($\chi = 0$). We obtain the slow-fast dynamical system for the normalized numbers of Krox20 proteins and mRNA

$$\begin{aligned} \partial_\tau \hat{m}(\tau) &= \chi\theta(\tau_1 - \tau) + f(\alpha\hat{n}) - \hat{m} \\ \partial_\tau \hat{n}(\tau) &= \frac{1}{\varepsilon}(\hat{m} - \hat{n}) \end{aligned} \tag{29}$$

3.1.1. Steady state analysis without Krox20 initiation

In Supplementary Figure S5C we plot the steady states (fixed points) for which $\hat{n}^* = \hat{m}^*$ (eq. 29) for $\chi = 0$ as function of α. We found that there is a minimal value $\alpha_{min} \approx 33.8$ such that for $\alpha < \alpha_{min}$ there is only one single fixed point at $\hat{n}^* = 0$ ($\alpha_{\min}$ can be computed using the solution z_0 of the equation $\frac{f'(x)}{f(x)} = \frac{1}{x}$, $\alpha_{min} = \frac{z_0}{f(z_0)} = \frac{1}{f'(z_0)}$). For $\alpha > \alpha_{min}$ we have a bistable system with two stable fixpoints $\hat{n}_1^*$ and $\hat{n}_3^*$ and a saddle point $\hat{n}_2^*$(see Supplementary Figure S5E). For large α, the asymptotic values $\hat{n}_2^* \to 0$ and $\hat{n}_3^* \to 0$ correspond to the situation where element A is fully activated, as can be seen from the steady state probabilities p_s for large α depicted in Supplementary Figure S5D.

The condition $\alpha > \alpha_{min}$ implies that a minimal *Krox20* mRNA production rate

$$\Phi_{A,min} = \alpha_{min} \frac{\psi\Psi}{\phi\beta} \tag{30}$$

is needed for generating a stable state where Krox20 is activated. In that case, there are two steady states, leading to a bistable equilibrium. Using the experimental finding that the heterozygous rate $\Phi_A = 0.18\ \text{min}^{-1}$ induces a high *Krox20* expression ((Voiculescu *et al*, 2001) and data not shown), the requirement $\Phi_A > \Phi_{A,min}$ results in a lower bound for the rate β:

$$\beta_{\min} = \frac{\alpha_{\min}}{n_0} \approx 0.1, \tag{31}$$

where we used $n_0 \approx 375$ (we used the values for Ψ, ψ and ϕ from Supplementary Table S1).

To analyze the impact of the initial production on the asymptotic behavior, we used the phase space: for $\alpha > \alpha_{min}$ there are two stable fixed points and a saddle point, and the separatrix passing through the saddle point separates the two basins of attractions associated with each stable points (see Supplementary Figure S5E for $\alpha = 40$). When the initiation is strong enough such that the initial conditions are outside the basin of attraction of $\hat{n}_1^* = 0$, it results in a high *Krox20* expression level. In contrast, if initiation is too weak, *Krox20* expression vanishes. In Supplementary Figure S5F we plot the separatrix for different values of α : when α increases, the basin of attraction of $\hat{n}_1^* = 0$ shrinks and asymptotically vanishes for large α, resulting in an exclusive high *Krox20* expression level

In summary, in the deterministic approximation, the dynamical system underlying *Krox20* activation is bistable for $\alpha > \alpha_{min}$. At the cellular level, this bistability is reflected in a binary cell-fate choice associated with a high *Krox20* level, or *Krox20* expression is not maintained. In the next paragraph we will investigate how this bistable cellular behaviour entails bimodality at the cell population level. The complete analysis requires accounting for the stochastic production of mRNA, which generates large discontinuous jumps in the number of produced mRNA and leads to large fluctuations in the number of Krox20 molecules.

3.2. Analysis of the stochastic model

To estimate the unknown ratio β, we numerically analyze equation 7 and evaluate the impact of initiation and autoregulation on the time evolution of the system. By simulating eq. 7, we obtain the probability $p(m,n,t)$ that fully describes the system. Although the simulations are performed with the variables m and n, we present the results using the scaled variables $\hat{m}$ and $\hat{n}^*$ to facilitate the comparison between the different scenarios. We use the notation $p(\hat{m},\hat{n},t)$ for $p(m,n,t)$. The marginal probabilities for Kox20 mRNA and proteins and the state of element A are defined in eq. 8. The time dependent relations for mean values for $\hat{m}$ and $\hat{n}$ are

$$\begin{aligned} \overline{\hat{m}}(t) &= \frac{1}{m_0}\sum_{m,n=0}^{\infty} mp(m,n,t) = \frac{1}{m_0}\sum_{m=0}^{\infty} mp(m,t) = \frac{\overline{m}(t)}{m_0}, \\ \overline{\hat{n}}(t) &= \frac{1}{n_0}\sum_{m,n=0}^{\infty} np(m,n,t) = \frac{1}{n_0}\sum_{n=0}^{\infty} np(n,t) = \frac{\overline{n}(t)}{n_0}, \end{aligned} \tag{32}$$

3.2.1. Impact of β on the production rate

In Supplementary Figure S6A-H we analyze *Krox20* activation up to a maximal time $t = 500\,\text{min}$ in heterozygous condition with $\Phi_A = 0.18\,\text{min}^{-1}$, for $\Phi_I = 0.7\Phi_A$ (endogenous situation, see eq. 15), $t_I = 80\,min$ and various β. By plotting the mean $\overline{\hat{m}}(t)$ and $\overline{\hat{n}}(t)$ as a function of time, we found that for the minimal value $\beta \approx 0.13$ the expression of *Krox20* is maintained (Supplementary Figure S6A,B). This corresponds to $\alpha \approx 50$ (see eq. 26), which is within the range of the minimal value $\alpha_{min} \approx 33.8$ for bistability. For $\beta > 0.13$, the probability distribution of Krox20 $p(\hat{n})$ at time $t = 500\,\text{min}$ (considered as steady state) is bimodal, with two peaks at zero and close to one (Supplementary Figure S6C). This bimodal distribution is a direct manifestation of bistability, which is induced by the intrinsic molecular fluctuations and is associated with two possible fates, Krox20-positive and -negative. We display in Supplementary Figure S6E-G the probability $p(\hat{n},t)$ for three values $\beta = 0.06$, $\beta = 0.13$ and $\beta = 0.4$. For $\beta = 0.06$, the strength of the initiation phase is not enough to activate element A, resulting in a fast decay of *Krox20,* which eventually vanishes (Supplementary Figure S6E). For $\beta = 0.13$, only a fraction of cells activate element A and a bimodal distribution emerges (Supplementary Figure S6F). Finally, for $\beta = 0.4$ almost all cells activate element A and the probability distribution becomes unimodal (Supplementary Figure S6G).

In Supplementary Figure S6D we plot the state probabilities p_s to find element A in state s at time $t = 500\,\text{min}$ as a function of β. The probabilities for the intermediate states *s*=1,2,3 are negligible, and only the states *s*=0 and *s*=4 reach large probabilities. The probability to find element A fully activated changes drastically in the range $\beta \in [0.1; 0.2]$, which is related to the critical value $\beta_{min} = 0.1$ found in the deterministic system.

3.2.2. Estimation of the ratio β

Experimentally we found that the level of *Krox20* expression in heterozygous and wild type embryos is very similar. To estimate β, we therefore constrained β such that the expression level in the heterozygous condition corresponds to at least 90% of the level in the wild type condition. In Supplementary Figure S6H we plot the ratio between the probability p_4 (probability of state s=4) to find element A fully activated at time $t = 500\,min$ in heterozygous and in wild type conditions as a function of β. Forβ> *0.20* the expression level in the heterozygous condition is more than 90% of the level in wild type condition, which gives the lower bound for β. For large β the expression levels in wild type and heterozygous conditions are large and robust against variations of β. However, we found that mouse rhombomere r3 is slightly smaller in heterozygous as compared to wild type embryos (Voiculescu *et al*, 2001), suggesting that β is close to the lower bound. We thus concluded

$$\beta \approx 0.20 \tag{33}$$

and we used this value in the following simulations.

3.2.3. A minimal mRNA production rate Φ_A is required to maintain Krox20 expression.

Previously we showed that a minimal value $\beta \approx 0.13$ is necessary to maintain *Krox20* expression for $\Phi_A = 0.18\ \text{min}^{-1}$ (Supplementary Figure S6A,B). For fixed $\beta = 0.20$, we searched for the minimal value of Φ_A required to maintain *Krox20* expression. In the deterministic system the minimal Φ_A is computed from eq.30. The precision of this relationship in the full stochastic system is however unclear. In Supplementary Figure S6I,J we show five simulations with $\beta = 0.20$, $\Phi_I = 2\Phi_A$ and $\alpha = (20,40,50,60,70)$ corresponding to $\Phi_A \approx (0.04,0.09,0.11,0.14,0.16)min^{-1}$. By choosing $\Phi_I = 2\Phi_A$ we ensured that Krox20 mRNA and protein levels induced by the initiation process exceed the steady state levels due to fully activated element A, such that a weak initiation cannot be responsible for the vanishing *Krox20* expression in Supplementary Figure S6I,J.

For $\Phi_A \approx 0.04\,min^{-1}$ ($\alpha = 20$)*, Krox20* expression is not maintained and quickly fades away after the initiation stops (Supplementary Figure S6I). For $\Phi_A \approx 0.09\,min^{-1}$ $\alpha = 40$), the expression declines at a slower pace. Finally, for $\Phi_A \approx 0.11\ \text{min}^{-1}$ (α = 50), *Krox20* expression is maintained, which is consistent with the minimal value for α found in the previous section when analyzing the impact of β. The rates $\Phi_A \approx 0.18\ \text{min}^{-1}$ (heterozygous) and $\Phi_A \approx 0.36\ \text{min}^{-1}$ (wild type) are largely above this minimum value and are therefore both sufficient to maintain a stable *Krox20* expression. Supplementary Figure S6J displays the probability distribution $p(\hat{n},t)$ for the number of Krox20 molecules at the end of the simulation and shows how bimodality emerges.

In the deterministic system we found that bistability occurs for $\alpha > 33.8$. By varying

Φ_A and β, we explored to which extent α can also be used in the Master equation to estimate the region where random switches occur. This region is approximately given by $\alpha > 50$. However, in contrast to the deterministic system, where the phase space changes abruptly for $\alpha = \alpha_{min}$, in the stochastic system, changes are smooth due to the impact of fluctuations.

3.2.4. Initiation gradually modulates the fraction of cells that express Krox20.

To analyze the impact of the initiation strength in wild type conditions, we computed the element A activation $p_s(t = 500\,\text{min})$ for $\Phi_A \approx 0.36\,\text{min}^{-1}$, $\beta = 0.20$ and $\Phi_I = \chi\Phi_A$ and various χ (Supplementary Figure S7A-D). We found that the maintenance of *Krox20* expression does not depend on Φ_I (Supplementary Figure S7A), but Φ_I gradually modulates the fraction of cells that adopt a Krox20-positive fate (Supplementary Figure S7B). This behavior is not captured by the deterministic dynamical system, where a sharp transition from zero to full activation occurs at a threshold value Φ_I, when the system crosses the separatrix and enters the basin of attraction of the large fixpoint. Hence the present stochastic analysis reveals that there is a gradual relationship between the level of *Krox20* initiation and the number of *Krox20*-positive cells.

The Krox20 protein probability distributions $p(\hat{n})$ at the end of the simulations have a bimodal shape with a peak at $\hat{n} = 0$ (*Krox20* is not expressed), and another near $\hat{n} = 1$, corresponding to cells with fully activated element A (Supplementary Figure S7C). Rescaling $p(\hat{n})$ by the peak value near $\hat{n} = 1$ further reveals that the distributions have identical shapes (Supplementary Figure S7D). Hence, varying the initiation strength only modulates the fraction of activated cells, but not the Krox20 distribution within these cells, showing that the initiation process does not introduce additional heterogeneity into the Krox20-positive cell population. The expression for $p(\hat{n})$ for $\hat{n} > 0$ can be expressed in the form $p(\hat{n}) = \zeta q(\hat{n})$, where ζ is the fraction of cells that adopt a *Krox20*-positive fate and $q(\hat{n})$ is the steady state probability distribution for the number of proteins in a system with mRNA and proteins and constant rates Φ_A, ϕ, Ψ and ψ. These distributions are not necessarily Poissonian, see (Thattai & van Oudenaarden, 2001; Bokes *et al*, 2012a).

Next we studied the effect of variations in the initiation time t_I in the wild type condition with $\Phi_A \approx 0.36\,\text{min}^{-1}$, $\beta = 0.20$ and $\Phi_I = 0.7\Phi_A$ (Supplementary Figure S7E-H). We found that changing the initiation time t_I is similar to changing the initiation strength Φ_I: the maintenance of *Krox20* expression does not depend on the value of t_I (Supplementary Figure S7E,F), t_I gradually modulates the fraction of cells that adopt a positive fate (Supplementary Figure S7G), and the bimodal distributions all have identical shapes (Supplementary Figure S7H). Hence, the initiation time and initiation strength similarly modulate the number of *Krox20*-positive cells.

Finally, we analyze how the initial conditions determine the cell fate. In the

deterministic approximation the position of the initial condition (number of $Krox20^I$ mRNA and proteins) in the phase space, relative to the separatrix, determines the cell fate (Supplementary Figure S5E,F). In the stochastic model the initial condition only determines the probability of the cell fate. Here the separatrix of the deterministic system is represented by a stochastic separatrix, which is given by the initial positions from where each fate is attained with probability ½(Schuss, 2010). In Supplementary Figure S7I,J, we plot the state probabilities of element A at time $t = 500\,min$ for two initial condition scenarios with zero initiation ($\Phi_I = 0$): in Supplementary Figure S7I we chose zero initial Krox20 proteins and we varied the initial number of mRNA molecules m; in Supplementary Figure S7J we vary the initial number of Krox20 proteins n with zero initial *Krox20* mRNA. Supplementary Figure S7I shows that the production of a single mRNA leads approximately 5% of the cells to the Krox20-positive fate, and 8 initial mRNA molecules almost fully activate element A. Hence, the transition occurs in a small range of mRNA molecules rendering the system very sensitive to changes in the number of mRNA molecules. In Supplementary Figure S7J we present the probability by which an initial number of Krox20 proteins induce a Krox20-positive cell fate, in which case the activation profile is determined by the sigmoid function $f(\beta n)$.

3.2.5. Variations in binding cooperativity, synergistic activation and number of binding sites

We explored the consequences of variations in the cooperativity coefficients $\tilde{\gamma}_i$, the synergy coefficients ξ_s and the number of binding sites. For fast binding and unbinding, $\tilde{\gamma}_i$ and ξ_s affect *Krox20* activation in a complex manner, which is revealed by the sigmoid function $f(\beta n)$ defined in eq. 28. The shape of $f(\beta n)$ (we used $\beta = 0.21$) as a function of the number of Krox20 proteins n determines the steady state and the sensitivity to initiation: the non-zero stable steady state of the deterministic model is given by the last intersection of $f(\beta n)$ with the line given by $\frac{n}{n_0}$, where $n_0 = \frac{\Phi_A}{\Psi}\frac{\phi}{\psi}$ (see eq. 26; dotted line in Supplementary Figure S8A,C); the sensitivity to initiation is reflected by the position of the saddle point (second intersection point).

We now discuss the impact on *Krox20* expression for a wild type situation with $\Phi_A = 0.36 min^{-1}$. We started by exploring changes in $f(\beta n)$ for various cooperativity coefficients $\tilde{\gamma}_i$. In Supplementary Figure S8A we compare $f(\beta n)$ computed with the wild type values $\tilde{\gamma}_i = \left(\frac{1}{378}, \frac{1}{4.3}, \frac{1}{14.1}, \frac{1}{3.3}\right)$ and $\xi_s = (0,0,0,0.23,1)$ (solid line) to cases without cooperativity, i.e. uniform coefficients $\tilde{\gamma}_i = \delta(1,1,1,1)$ (dashed lines), where δ is a scaling factor that modulates the affinity of the four Krox20 binding sites. For low δ ($\delta = \frac{1}{50}$), the non-zero steady state vanishes (no intersection in Supplementary Figure S8A, and no separatrix in Supplementary Figure S8B) and Φ_A is not strong enough to maintain *Krox20* expression. With $\delta = \frac{1}{3}$, the system is extremely sensitive and a small initiation level can activate element A (Supplementary Figure S8B, red line). A system similar to the wild type situation can be reconstituted with an

intermediate value $\delta = \frac{1}{15}$. In this case, even without cooperativity, the sigmoid shape of $f(\beta n)$ is conserved (Supplementary Figure S8A) and the separatrix is similar to the wild type situation (Supplementary Figure S8B). However, the efficiency of activation is reduced (Supplementary Figure S8A).

We then explored the consequences of altering the synergy (Supplementary Figure S8C): the no-synergy scenario was computed with $xi_s = (0,1,1,1,1)$. When binding cooperativity is maintained, the sigmoid shape of $f(\beta n)$ is largely unaffected (Supplementary Figure S8C, compare black and blue curves). However, without synergy the activation become more sensitive to low numbers of Krox20 protein and therefore to low levels of initiation. In absence of both synergy and cooperativity (in this case, the no-cooperativity scenario was computed with $\tilde{\gamma}_i = \frac{1}{15}(1,1,1,1)$), the sigmoid shape is lost and the activation becomes extremely sensitive to initiation (Supplementary Figure S8C, green curve). This result shows that synergy is absolutely required to maintain a sigmoid shape when binding cooperativity is abolished. This can be understood by considering the impact of the intermediate states (s=1,2,3). With binding cooperativity, element A is either fully activated or fully deactivated (see Supplementary Figure S8D, with cooperativity and without synergy) and the impact of the intermediate states remains small independent of the synergy. In contrast, in absence of binding cooperativity and synergy, the intermediate states attain relatively large probabilities (Supplementary Figure S8E); the system therefore loses its bistability.

Finally, changing the number of binding sites, by eliminating one of them operating with a high synergy has a dramatic impact on the feedback loop. Indeed, the system is then unable to maintain *Krox20* expression because the maximal activation state of the promoter ($\Phi_{A,4}$) is lost (Supplementary Figure S8F, red curve; see also the discussion in section 3.2.3). Hence, masking one of the binding sites, e.g. through competitive binding, might constitute an efficient mechanism to abolish previously established *Krox20* expression. Without synergy, lowering the number of binding sites only reduces the efficiency of activation (Supplementary Figure S8G).

To conclude, binding cooperativity and synergy are both required to shape the sigmoidal *Krox20* activation curve. Loss of either of them may be compensated by increasing the affinity of the binding sites for Krox20 or the efficiency of transcription machinery recruitment. These compensations would occur at the expense of an increased sensitivity to low initiation inputs, making the Krox20 feedback loop much more sensitive to fluctuations.

Supplementary Figure S1

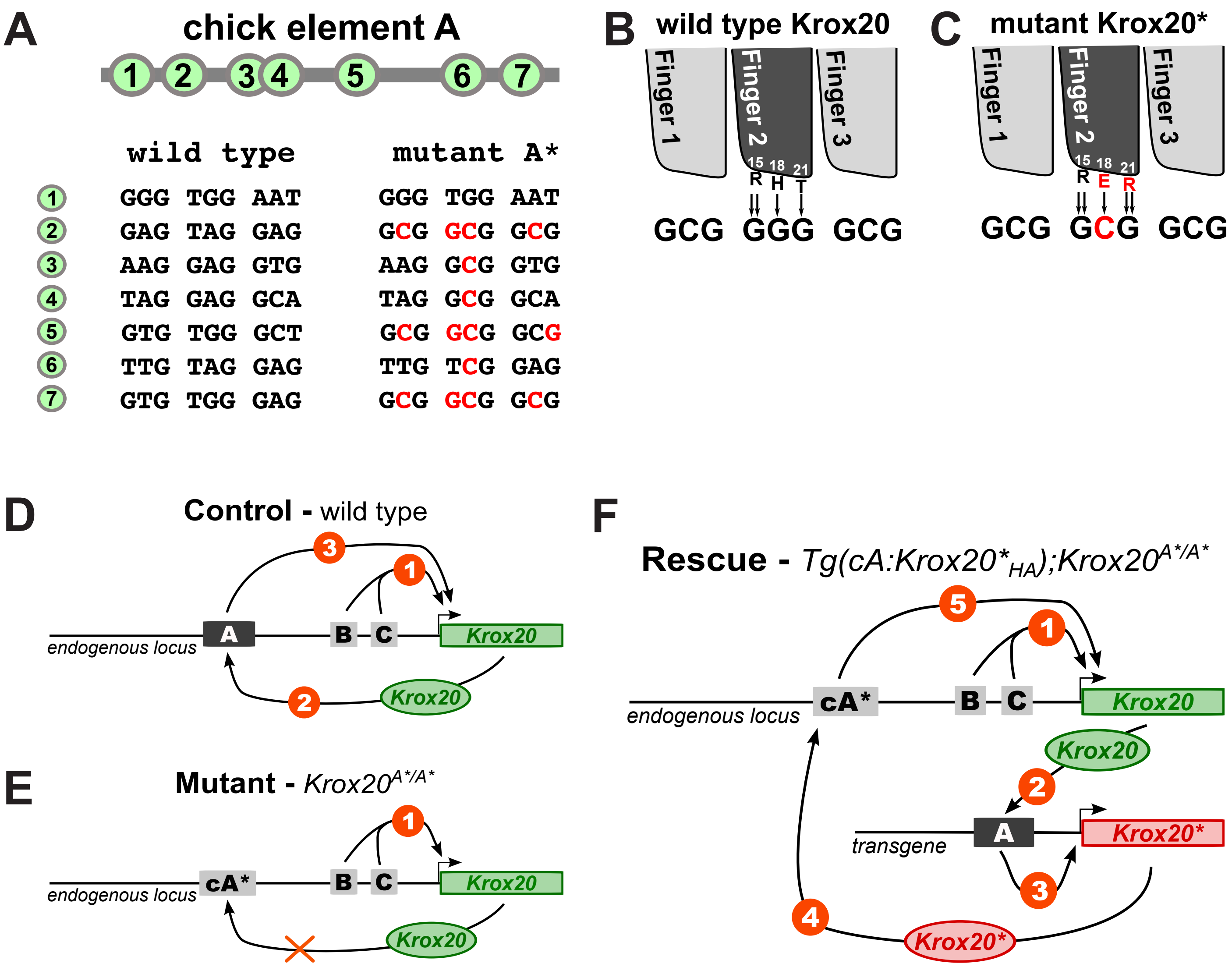

Supplementary Figure S1 (related to Figure 1). Krox20 DNA binding specificity and design of the autoregulatory rescue.

(A) Positions of the seven putative Krox20 binding sites along element A and their nucleotide sequences in either their wild type element A or mutant element A*. Modified nucleotides in the mutant version are indicated in red.

(B) Schematic representation of the interactions of wild type Krox20 and mutant Krox20* with their respective consensus binding sites. Modified amino acids and nucleotides in the mutant versions are indicated in red.

(D)Schematic representation of the autoregulatory loop in the wild type situation:*Krox20* expression involves two phases, initiation (1) and autoregulation (2,3).

(E) In the *Krox20*$^{A*/A*}$ mutant, the autoregulation phase is abolished since element A is replaced by the mutantelement A*.

(F) The*Krox20*$^{A*/A*}$mutant can theoretically be rescued by providing a transgene that expresses*Krox20** in r3 and r5. In this situation, *Krox20* is normally initiated (1) and activates the exogenous element A driving expression of *Krox20**(2,3); in turn Krox20* activates A* on the endogenous locus (4), leading to further production of Krox20 (5). The rescue is based on a novel, indirect autoregulatory loop.

Supplementary Figure S2

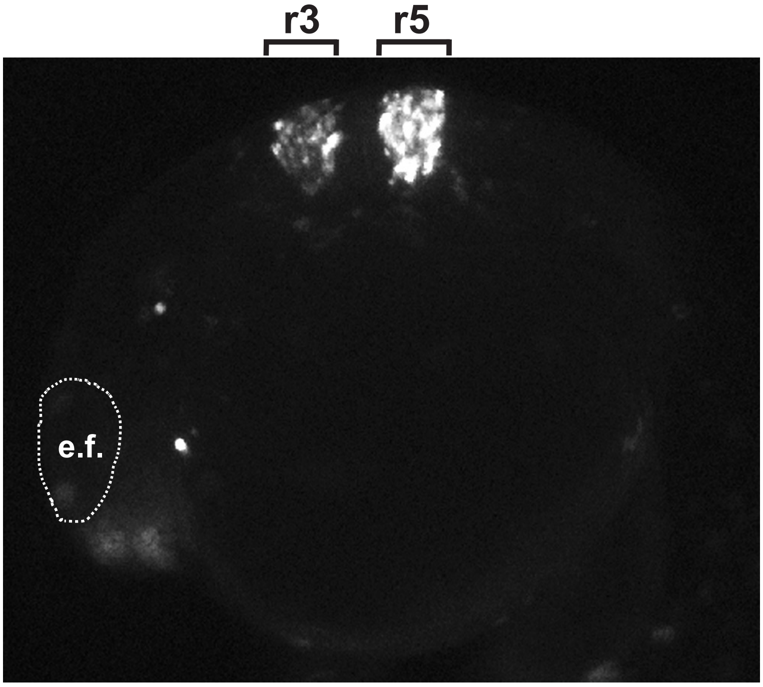

Supplementary Figure S2 (related to Figure 2). The putative zebrafish element A drives specific expression in r3 and r5.

A pTol2 construct carrying the *gfp* gene under the control of the candidate sequence for the zebrafish element A was injected into zebrafish embryos at the 1 cell-stage. Analysis of the fluorescence in 20-somite embryos shows expression of *gfp* restricted to r3 and r5. e.f. : eye field.

Supplementary Figure S3

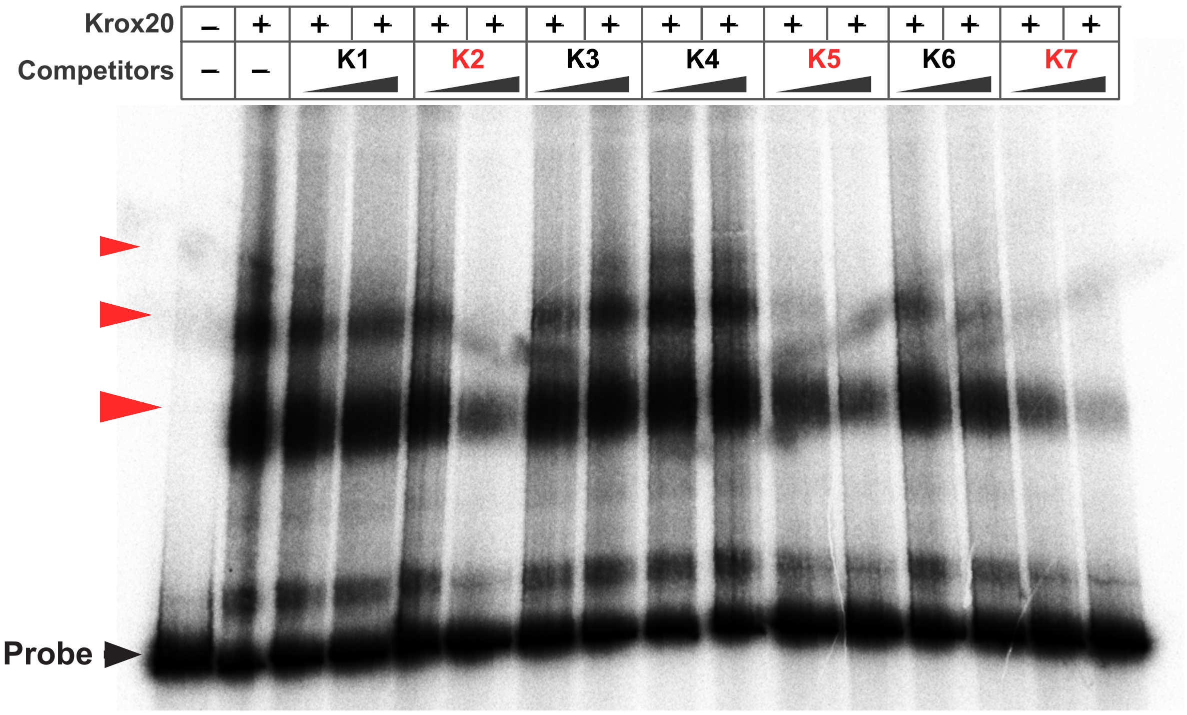

Supplementary Figure S3 (related to Figure 3). Identification of bona fide Krox20 binding sites in element A.

A radiolabelled DNA probe (25 nM) corresponding to element A was subjected to EMSA in presence of bacterial extracts containing Krox20 and cold competitor oligonucleotides carrying the seven putative Krox20 binding sites identified in silico. Three binding complexes are observed in these conditions (red arrows), suggesting that the element A carries at least three sites. The competitors are provided at two concentrations (25 and 100 nM). Oligonucleotides corresponding to sites 2, 5 and 7 severely affect the binding of Krox20 on element A, whereas the site 6 oligonucleotide competes less efficiently. The other oligonucleotides do not compete. The sequences of the competitor oligonucleotides are presented in Supplementary Table S2B.

Supplementary Figure S4

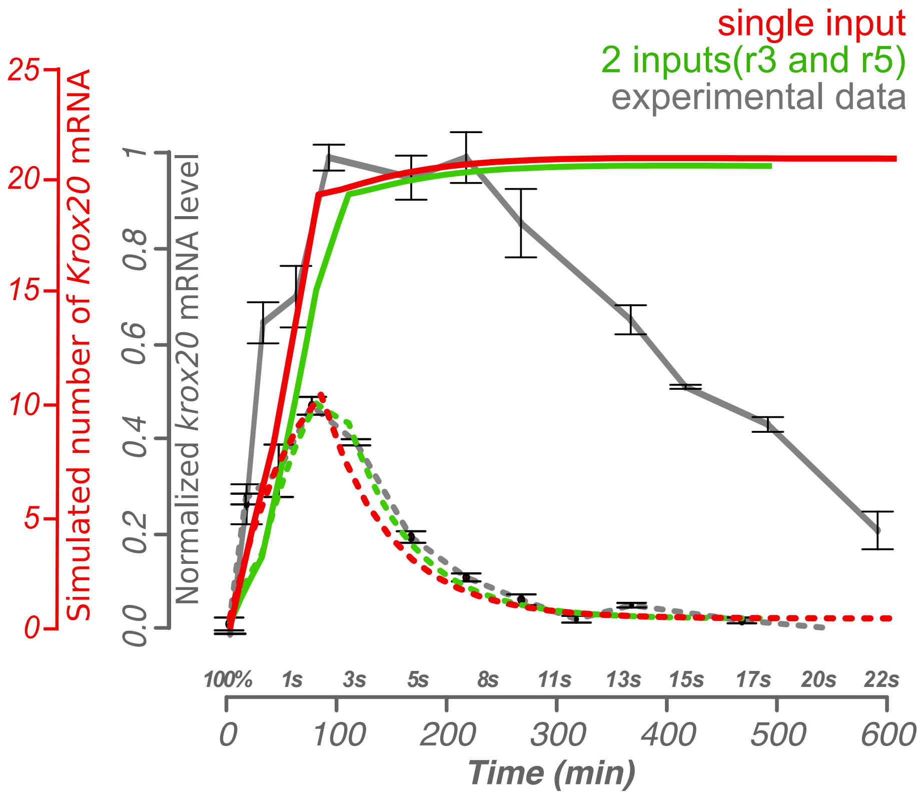

Supplementary Figure S4 (related to Figure 4B). Analysis of the effect of shifted initiation inputs in r3 and r5.

Comparison of the simulations obtained with a single input (red) or with two shifted inputs of the same Φ_I value for r3 and r5 (green), with the experimental data (grey) of *Krox20* expression time courses in developing embryos. Solid and dashed curves correspond to simulations and measures with and without activity of the autoregulatory loop, respectively. In the simulation with two inputs, the first one initiates at t=0 and lasts for 80 min and the second one initiates at t=10 min and lasts for 130 min.

Supplementary Figure S5

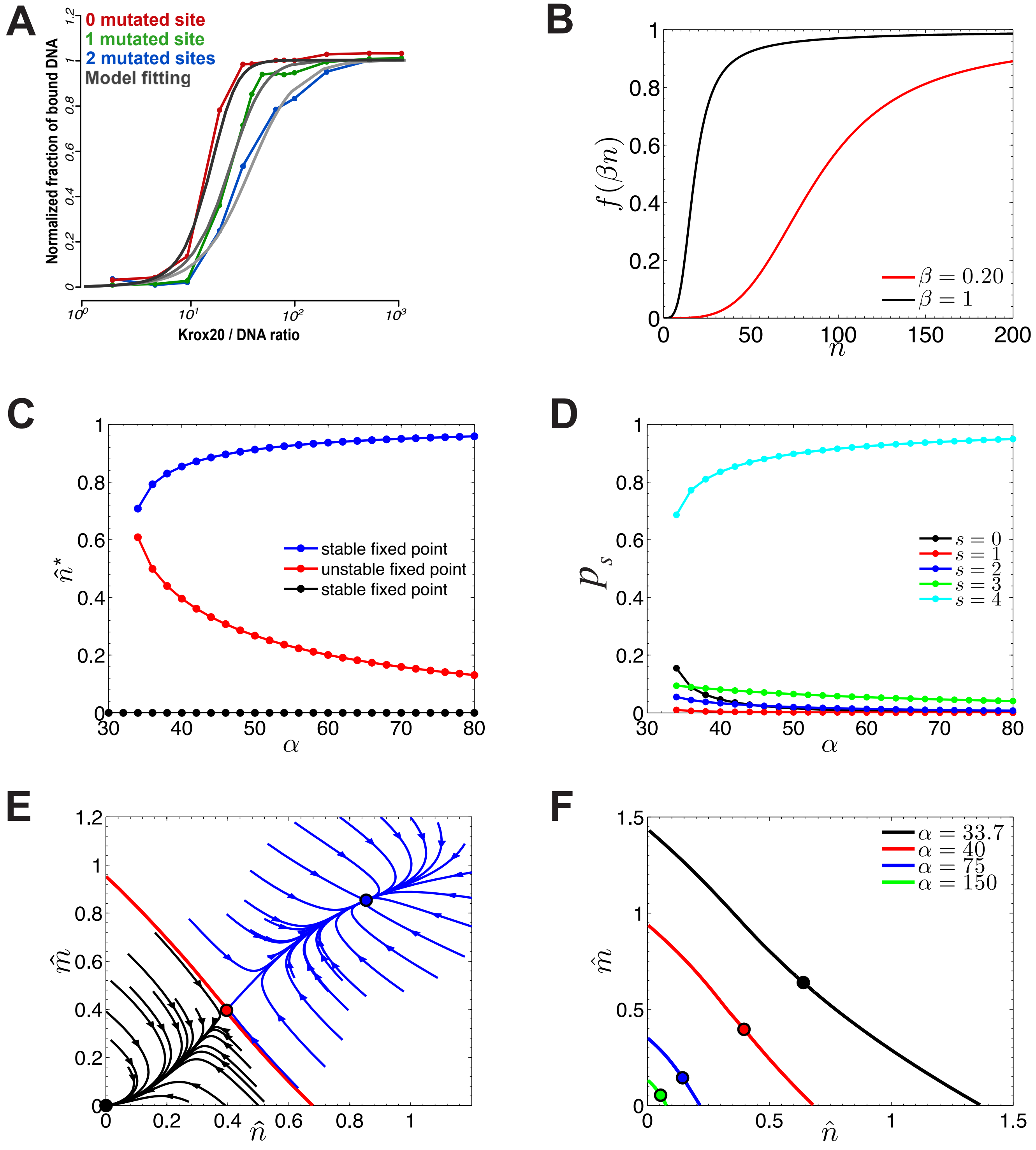

Supplementary Figure S5 (related to ST, sections 2 and 3). Analysis of binding cooperativity and of the deterministic dynamical system

(A) Experimental curves for the ratio of bound Krox20 proteins (colored curves). These curves were fitted using ST eq. 21 for N_b=4,3,2 to extract the values of $\tilde{\gamma}$ (see ST for further details).

(B) The sigmoid function $f(\beta n)$ defined in ST eq. 28 as a function of the number of Krox20 proteins n for $\beta = 1$ and $\beta = 0.20$.

(C) Fixpoints $\hat{m}^* = \hat{n}^*$ of eq. 40 (with $\chi = 0$) as a function of the critical parameter α ($\hat{m}$ and $\hat{n}$ are the scaled values of m, number of *Krox20* mRNA, and n, respectively). The black and blue points are the stable fixpoints $\hat{n}_1^* = 0$ and $\hat{n}_3^*$, whereas the red points are the saddle points $\hat{n}_2^*$. Bistability is achieved for $\alpha > \alpha_{\min} \approx 33.8$.

(D) Steady state probabilities $p_s(n)$ defined in ST eq. 4 of finding element A in state s at the stable fixpoint $\hat{n}_3^*$.

(E) Phase space analysis. The two stable fixpoints $\hat{n}_1^* = 0$ and $\hat{n}_3^*$ are indicated in black and blue respectively, and the saddle point $\hat{n}_3^*$ is indicated in red. The blue trajectories converge versus the large fixpoint, whereas Krox20 expression eventually vanishes along the black trajectories. The red curve is the separatrix.

(F) Separatrix for various values of α. The basin of attraction of the fixpoint $\hat{m}^* = \hat{n}^* = 0$ shrinks with increasing α.

Supplementary Figure S6

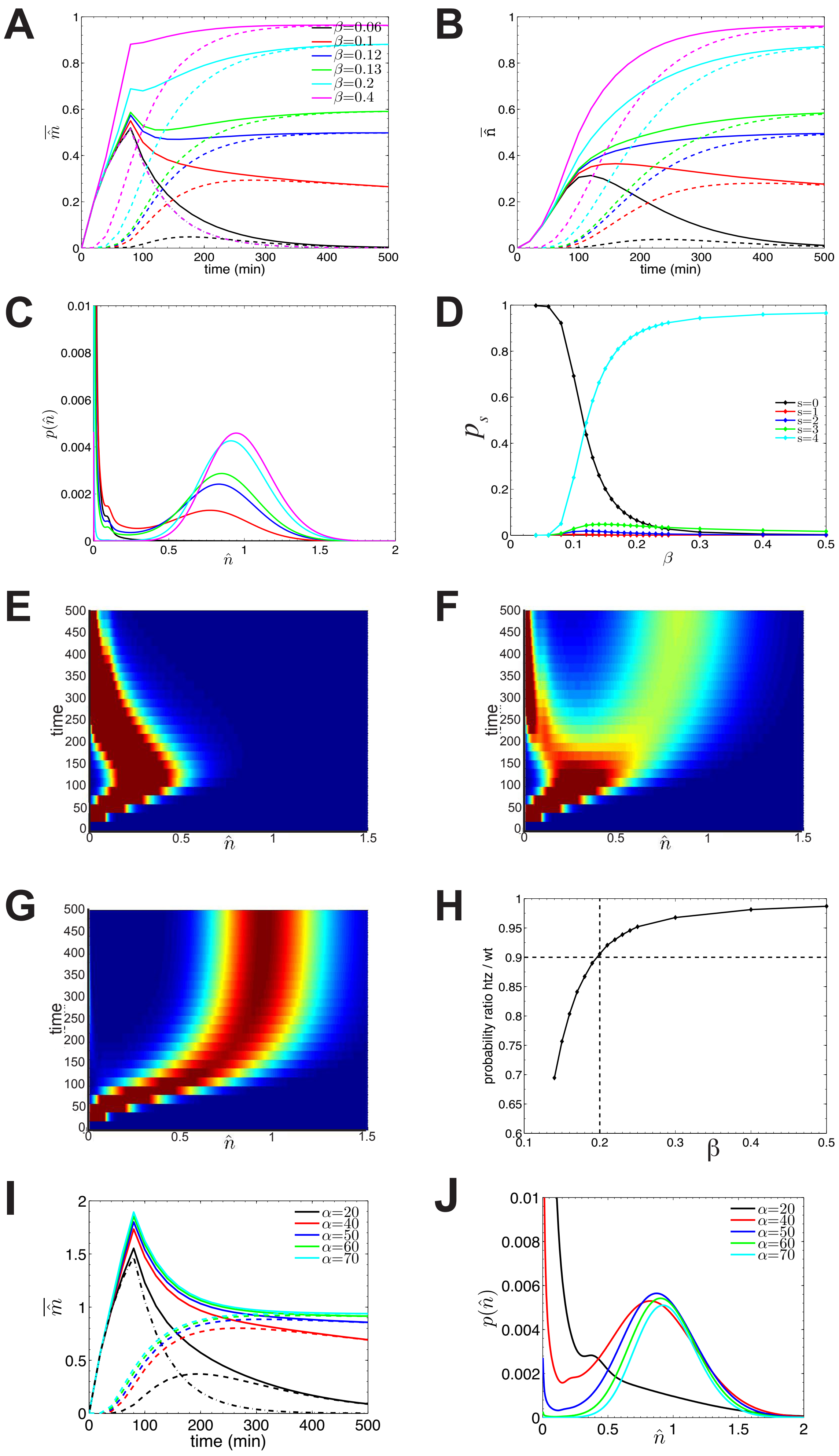

Supplementary Figure S6 (related to ST, section 3). Analysis of the stochastic system and determination of the value of the parameter β.

The results shown in panels (A-H) are obtained from simulations of ST eq. 7 with the heterozygous production rate $\Phi_A = 0.18\ \text{min}^{-1}$, $\Phi_I = 0.7\Phi_A$, $t_I = 80$ min and various β. The simulations in (I,J) are obtained with $\Phi_I = 2\Phi_A$, $t_I = 80$ min, $\beta = 0.20$ and various $\Phi_A \approx (0.04, 0.09, 0.11, 0.14, 0.16) min^{-1}$ corresponding to $\alpha = (20,40,50,60,70)$ (see ST eq. 26).

(A) Time evolution of the mean number of *Krox20* mRNA molecules. The solid lines represent the total number of mRNA molecules, the dashed lines the number of mRNA produced by element A, and the dashed-dotted line the number of mRNA molecules produced by initiation.

(B) Mean number of Kox20 proteins with the same color code as in A.

(C) Bimodal probability distribution of the number of Krox20 proteins at the end of the simulation with the color code from A.

(D) Probabilities p_s to find element A in state s at the end of the simulation. (E-G) Heat-maps of the time evolution of the probability distributions $p(\hat{n},t)$ (densities) for $\beta = 0.06$, $\beta = 0.13$ and $\beta = 0.4$. Red color represents a high probability, blue a low probability.

(H) Ratio of the probabilities p_4 at time $t_I = 500$ min in heterozygous ($\Phi_A = 0.18\ \text{min}^{-1}$) and wild type condition ($\Phi_A = 0.36 min^{-1}$) as a function of β. The probability p_4 in the heterozygous condition is shown in panel D.

(I) Time evolution of the mean number of *Krox20* mRNA molecules for various Φ_A. The solid lines represent the total number of mRNA molecules, the dashed lines the number of mRNA produced by element A, and the dashed-dotted line the number of mRNA molecules produced by initiation(due to the scaling and $\Phi_I = 2\Phi_A$, the initiation curves does not change with Φ_A).

(J) Probability distribution of the number of Krox20 proteins at the end of the simulation.

Supplementary Figure S7

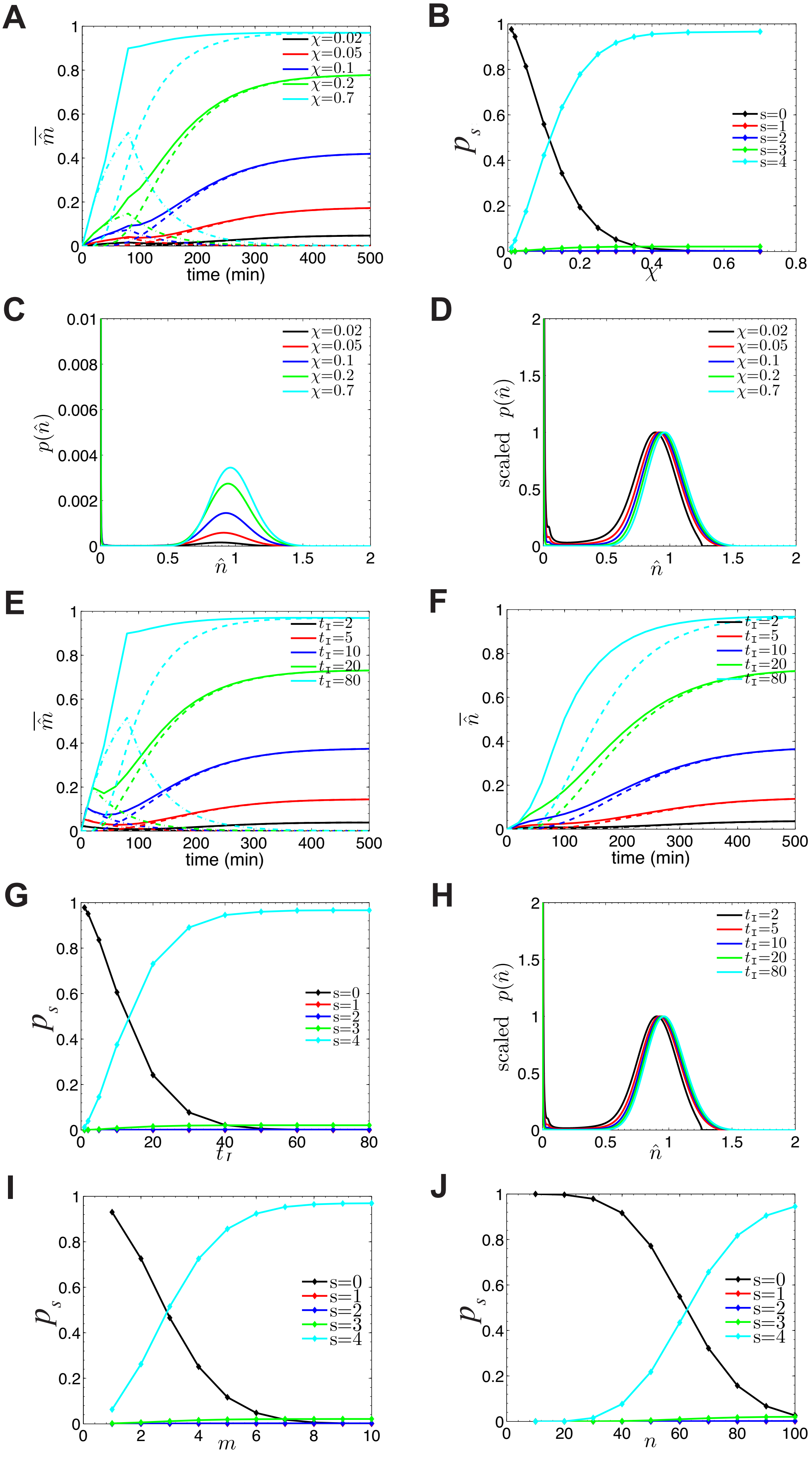

Supplementary Figure S7 (related to ST, section 3). Analysis of the initiation process and of the initial condition in wild type situation.

(A-D) **Analysis of the initiation strength**. The figures display results obtained from simulations of ST eq. 7 for the wild type production rate $\Phi_A = 0.36\ \text{min}^{-1}$, $t_I = 80$ min , $\beta = 0.20$, $\Phi_I = \chi\Phi_A$ and various χ.

(A) Time evolution of the mean numbers of *Krox20* mRNA molecules. The solid lines are the total numbers of mRNA molecules, the dashed lines the numbers of mRNA produced by element A, and the dashed-dotted lines the numbers of mRNA produced by initiation.

(B) Probability to find element A in state s at the end of the simulation.

(C) Probability distribution of the numbers of Krox20 proteins at the end of the simulation with the same color code as in A.

(D) Probability distributions from C rescaled by their peak values.

(E-H) **Analysis of the initiation time**. The figures display results obtained with $\Phi_A = 0.36\ \text{min}^{-1}$, $\beta = 0.20$, $\Phi_I = 0.7\Phi_A$ and various t_I.

(E) Time evolution of the mean number of *Krox20* mRNA molecules.

(F) Time evolution of the mean numbers of Krox20 proteins.

(G) Probability to find element A in state s at the end of the simulation.

(H) Probability distribution of the number of Krox20 proteins at the end of the simulation rescaled by their peak values.

(I-J) **Analysis of the initial condition.** The figures displays the state probabilities p_s at time $t_I = 500$ min obtained with $\Phi_I = 0$, $\Phi_A = 0.36\ \text{min}^{-1}$.

(I) Variations the initial number of mRNA molecules m with zero initial Krox20 proteins $n = 0$.

(J) Variation of the initial number of Krox20 proteins n with zero initial Krox20 mRNA $m = 0$.

Supplementary Figure S8

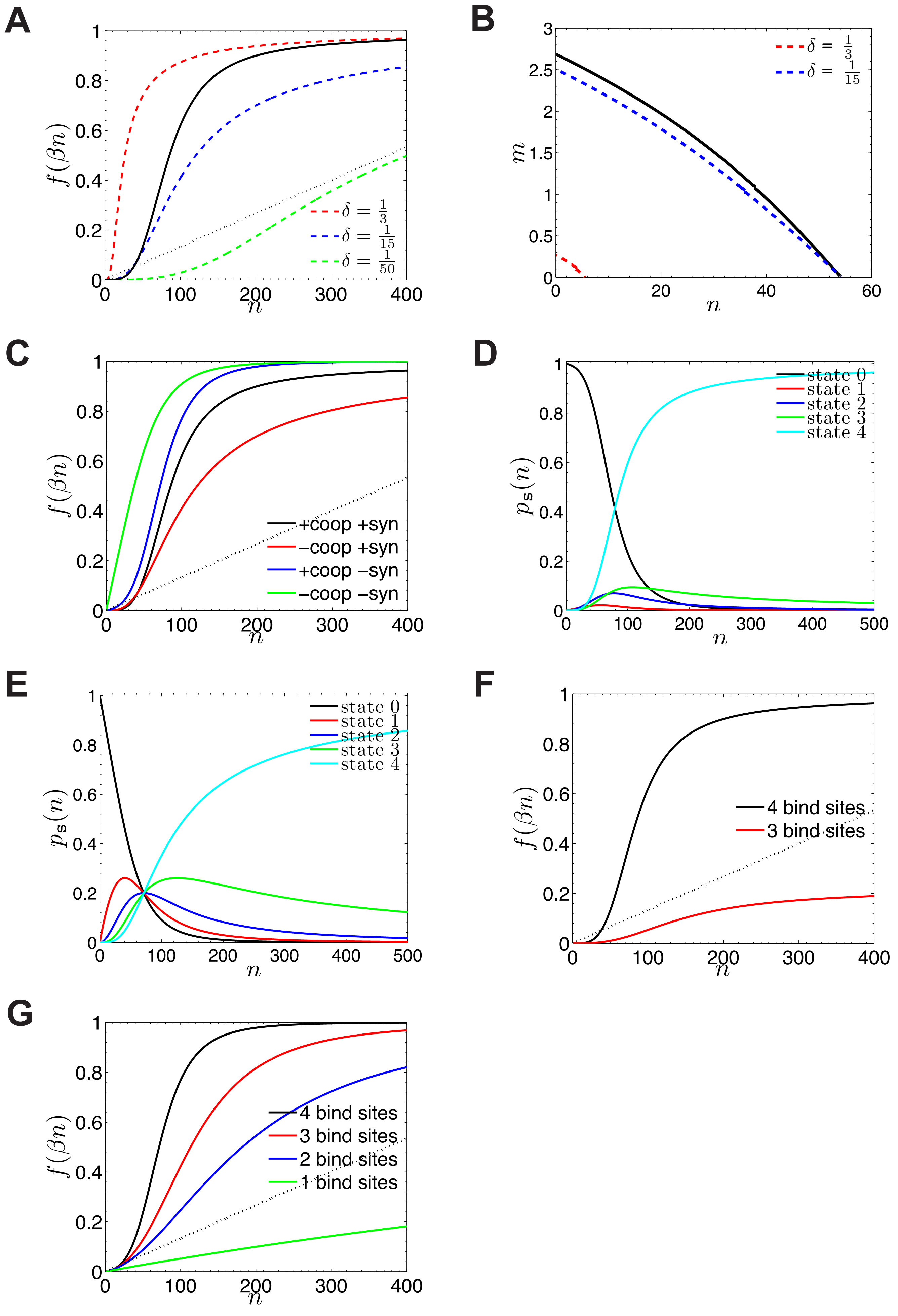

Supplementary Figure S8 (related to ST, section 3). Variation of the cooperativity coefficients $\tilde{\gamma}_i$, the synergy coefficients ξ_s and the number of Krox20binding sites.

(A) Comparison of $f(\beta n)$ computed from eq. 38 ($\beta = 0.20$) with wild type $\tilde{\gamma}_i = (\frac{1}{378}, \frac{1}{4.3}, \frac{1}{14.1}, \frac{1}{3.3})$ and $\xi_s = (0,0,0,0.23,1)$ (black solid line), in situations of no cooperativity modeled with $\tilde{\gamma}_i = \delta(1,1,1,1)$ (dashed lines). The intersections with the dotted line are the steady states of the deterministic model.

(B) Separatrix for the situations depicted in A.

(C) Comparison of $f(\beta n)$ in presence or absence of cooperativity and/or synergy. The no-cooperativity situation is computed with $\tilde{\gamma}_i = \frac{1}{15}(1,1,1,1)$ and the no-synergy with $\xi_s = (0,1,1,1,1)$.

(D) Steady state probability to find element A in state s (ST eq. 4) obtained from a simulation with cooperativity but no synergy.

(E) Steady state probability obtained from a simulation with no cooperativity and no synergy.

(F) Comparison of $f(\beta n)$ computed with 4 (black line) and 3 (red line) element A binding sites (with cooperativity and synergy).

(G) Comparison of $f(\beta n)$ for various number of element A binding sites with cooperativity and no synergy.

Supplementary Figure S9

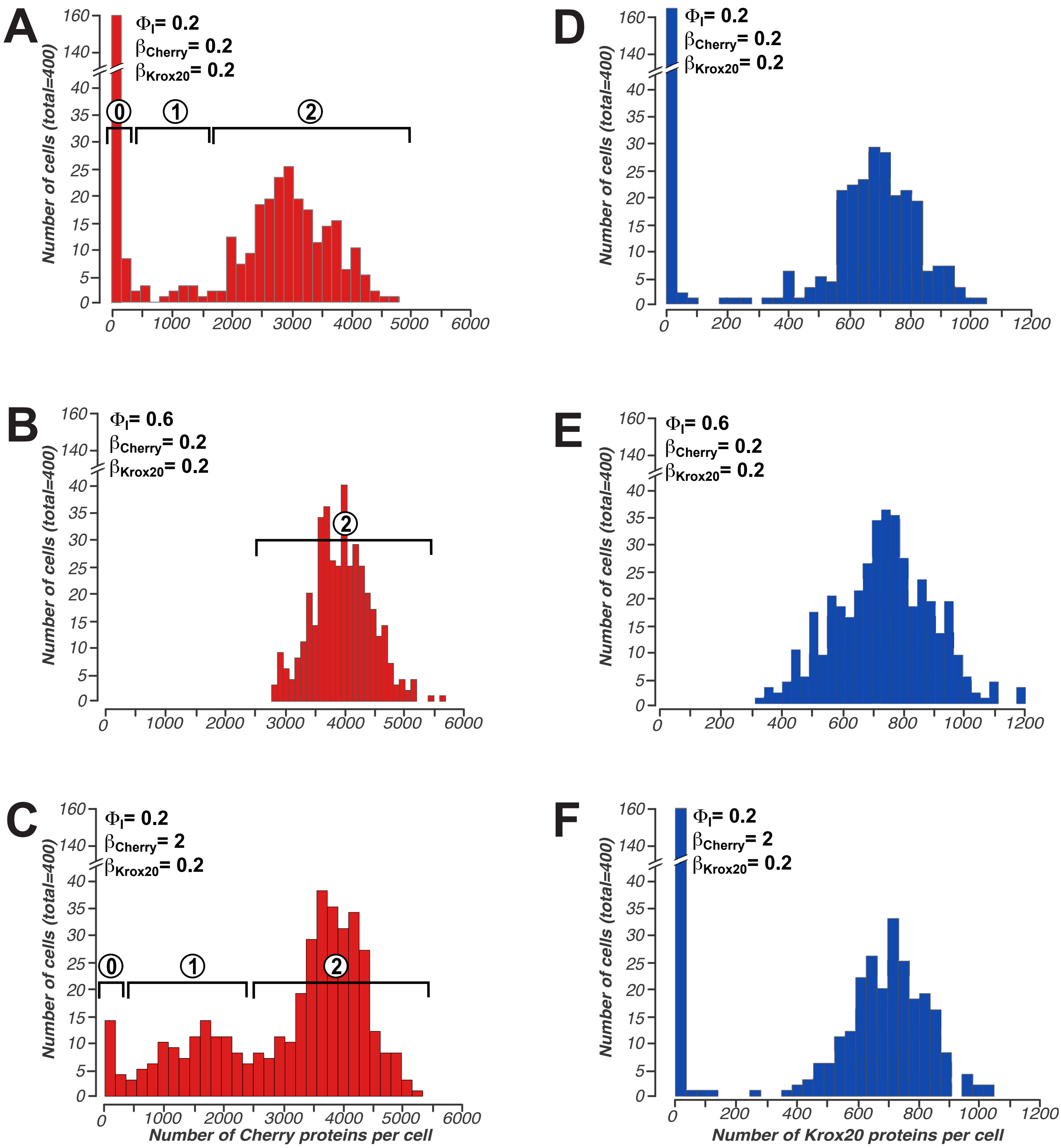

Supplementary Figure S9 (related to Figure 6). Simulation of the bimodal cell distribution observed with the reporter gene in even-numbered rhombomeres.

We modified the model to introduce the *Tg(cA:h2b-cherry)* transgene, and to take into account the stability of the Cherry and the possible different accessibilities of element A in the transgene and in its endogenous location. (A-C) Numerical simulations of the cell distributions based on the number of Cherry proteins were performed with the indicated parameters: Φ_I is the initiation production rate of *krox20* mRNA, β_{Cherry} and β_{Krox20} reflect the different accessibilities of Krox20 to exogenous (in the *Tg(cA:h2b-mcherry)* transgene) and endogenous element A, respectively. (D-F) Simulations of the cell distributions according to the number of Krox20 proteins, performed with the indicated parameters (same as in A-C, respectively). Three types of cell populations are observed with the following characteristics. 0: no expression of Cherry or Krox20; 1: moderate-level expression of Cherry and no expression of Krox20; 2: high-level expression of both Cherry and Krox20. The simulations reproduce two features observed experimentally. i) Population 2, which corresponds to experimental peak 2 in Fig. 6H, is translated towards higher levels of Cherry upon increasing initiation level Φ_I (compare A and B), whereas the position of the Krox20 high expression peak is unaffected (compare D with E). The shift of population 2 results from the high stability of Cherry compared to Krox20, which leads to accumulation of Cherry; ii) population 1, which corresponds to experimental peak 1 in Fig. 6H, comprises cells that have accumulated Cherry without successful activation of the Krox20 autoregulatory loop. This situation happens when the parameter β_{Cherry} is higher than β_{Krox20}, reflecting a better accessibility of Krox20 to element A in the transgenic configuration compared to the endogenous locus. This effect is further amplified by the stability of the Cherry protein.

Supplementary Figure S10

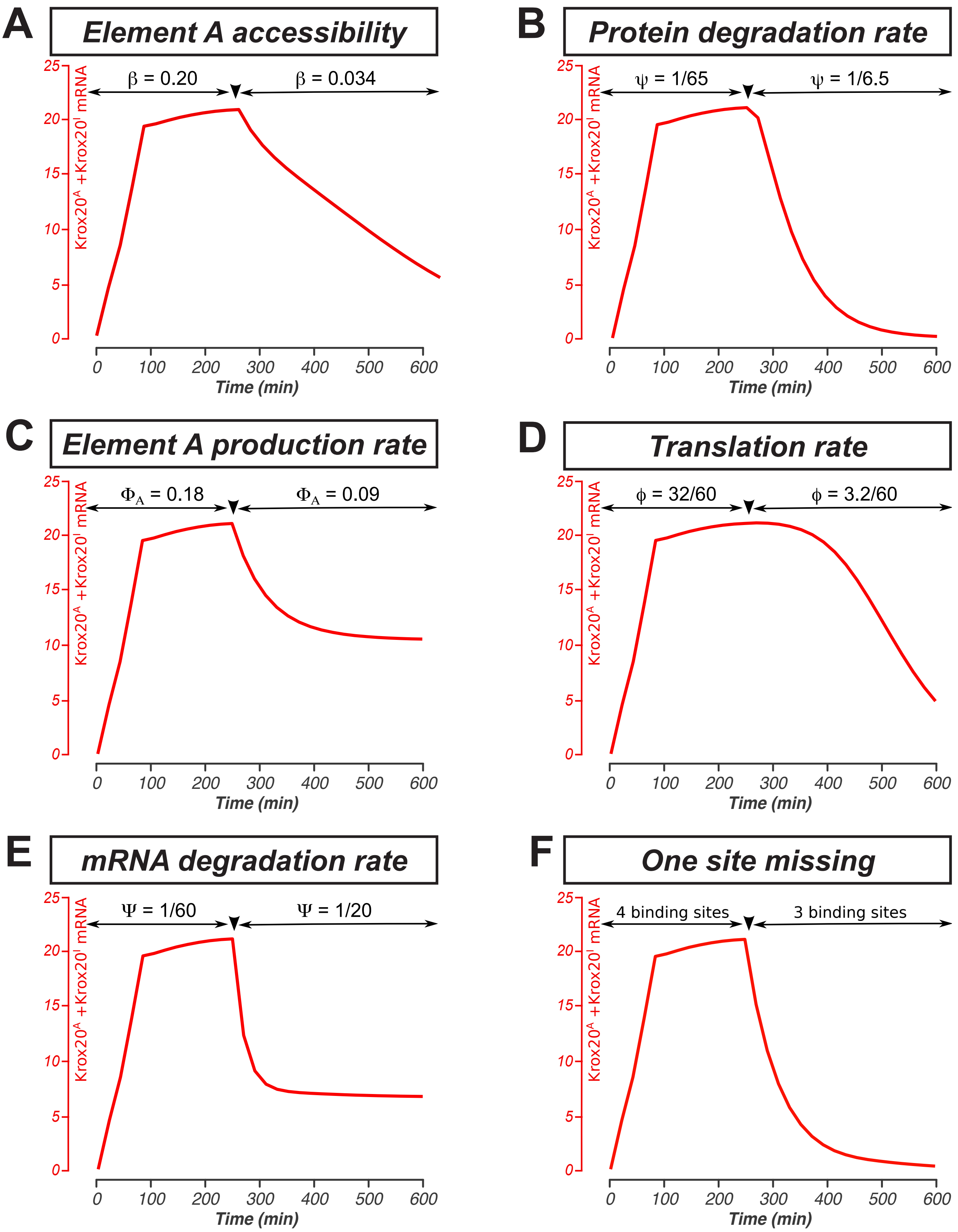

Supplementary Figure S10 (related to Figure 7A). Scenarios for the decay of *Krox20* expression.

(A-F) Simulations of the time evolution of the level of *Krox20* mRNA following a stimulation with the same parameters as Fig. 4A, but with the indicated modification of a specific parameter at t = 250 min. Modification of β is the only one leading to a linear-like decrease as observed experimentally (see also Fig. 7A).

Supplementary Table S1

A

Variable	Symbol
States of element A	s=[0, 1, 2, 3, 4]
Number of *Krox20* mRNA produced	m
Number of Krox20 proteins produced	n

B

Parameter	Symbol	Unit	Estimated/Fitted value (endogenous situation)
Number of Krox20-binding sites			4
Fractional binding rate	$\lambda_s = [\lambda_1, \lambda_2, \lambda_3, \lambda_4, 0]$ $= \lambda.[1, 1, 1, 1, 0]$	min^{-1}	1. [1, 1, 1, 1, 0]
Fractional unbinding rate	$\mu_s = [0, \mu_1, \mu_2, \mu_3, \mu_4]$ $= \lambda/\beta.[0, 1/\gamma_1, 1/\gamma_2, 1/\gamma_3, 1/\gamma_4]$	min^{-1}	1/0.2 [0, 1/378, 1/4.3, 1/14.1, 1/3.3]
Production rate during initiation	Φ_I	mRNA/min/allele	0.13
Duration of initiation phase	t_I	min	80
Fractional production rate of A	$\Phi_{A,s} = \Phi_A .\xi_s$	mRNA/min/allele	0.18 [0, 0, 0, 0.23, 1]
Translation rate	ϕ	protein/mRNA/min	0.57
Degradation rate of Krox20 mRNA	Ψ	min^{-1}	1/65
Degradation rate of Krox20 proteins	ψ	min^{-1}	1/60

(A) Variables of the model.
(B) Parameters of the model.

Supplementary Table S2

A

**Allele**		**PCR Primers**
Krox20^{NA*AK}	F	5' ACGAATGTCTATTTGTAGGTCCCAGGC 3'
	R	5' CAACCACGCTCAATGTTTTC 3'
Krox20^{A*}	F	5' CGCAGTGCCGTCCTCAAAGAGA 3'
	R	5' CAACCACGCTCAATGTTTTC 3'
Krox20^{NA}	F	5' GTAGAAGGTGGCGCGAAGGGGC 3'
	R	5' CCACACTGGAAGCTCGGGTATTG 3'
Krox20$^{\Delta A}$	F	5' GCGAGTTTCCTTGAAAGGAGC 3'
	R	5' CAACCACGCTCAATGTTTTC 3'
*cA::Krox20**$_{HA}$	F	5' ATTTGCTCCTCGCACACC 3'
	R	5' CTGAGAAGCCTGTCTTTAACTACTG 3'
r2 HPAP	F	5' GAGCGCAGCCTTCCAGAAGC 3'
	R	5' TTGACCCCGCACAGGTAGGC 3'

B

**Site**	**Competitor oligonucleotides**
**K1**	5'-CTCTGTACAAGGGTGGAGTTA-3'
**K2**	5’-CTCTGTACGAGTAGGAGGTTA-3’
**K3**	5'-CTCTGTACAAGGAGGTGGTTA-3'
**K4**	5'-CTCTGTACTAGGAGGCAGTTA-3'
**K5**	5’-CTCTGTACGTGTGGGCTGTTA-3’
**K6**	5'-CTCTGTACTTGTAGGAGGTTA-3'
**K7**	5’-CTCTGTACGTGTGGGAGGTTA-3’

(A) Sequences of the PCR primers used for genotyping the indicated alleles. (B) Sequences of the oligonucleotide competitors used in the EMSA experiments. The putative Krox20 binding sites are underlined.

Supplementary References

Bokes P, King JR, Wood ATA & Loose M (2012a) Exact and approximate distributions of protein and mRNA levels in the low-copy regime of gene expression. *J Math Biol* **64:** 829–854

Bokes P, King JR, Wood ATA & Loose M (2012b) Multiscale stochastic modelling of gene expression. *J Math Biol* **65:** 493–520

Gillespie DT (2005) A general method for numerically simulating the stochastic time evolution of coupled chemical reactions. *Journal of computational physics*

Gunawardena J (2005) Multisite protein phosphorylation makes a good threshold but can be a poor switch. *Proc Natl Acad Sci USA* **102:** 14617–14622

Ramos AF, Innocentini GCP & Hornos JEM (2011) Exact time-dependent solutions for a self-regulating gene. *Phys Rev E Stat Nonlin Soft Matter Phys* **83:** 062902

Reingruber J, Abad E & Holcman D (2009) Narrow escape time to a structured target located on the boundary of a microdomain. *J Chem Phys* **130:** 094909

Schuss Z (1980) Theory and applications of stochastic differential equations John Wiley & Sons Inc

Schuss Z (2010) Theory and applications of stochastic processes.

Schuss Z, Singer A & Holcman D (2007) The narrow escape problem for diffusion in cellular microdomains. *Proc Natl Acad Sci USA* **104:** 16098–16103

Schwanhäusser B, Busse D, Li N, Dittmar G, Schuchhardt J, Wolf J, Chen W & Selbach M (2011) Global quantification of mammalian gene expression control. *Nature* **473:** 337–342

Schwanhäusser B, Busse D, Li N, Dittmar G, Schuchhardt J, Wolf J, Chen W & Selbach M (2013) Corrigendum: Global quantification of mammalian gene expression control. *Nature* **495:** 126–127

Sidje RB (1998) Expokit: a software package for computing matrix exponentials. *ACM Transactions on Mathematical Software (TOMS)*

Thattai M & van Oudenaarden A (2001) Intrinsic noise in gene regulatory networks. *Proc Natl Acad Sci USA* **98:** 8614–8619

Voiculescu O, Taillebourg E, Pujades C, Kress C, Buart S, Charnay P & Schneider-Maunoury S (2001) Hindbrain patterning: Krox20 couples segmentation and specification of regional identity. *Development* **128:** 4967–4978
