# Supplementary material for: Dissection of a Krox20 positive feedback loop driving cell fate choices in hindbrain patterning
Source: Mol Syst Biol. 2013 Sep 24;9:690. doi: 10.1038/msb.2013.46 (PMC3792346; doi:10.1038/msb.2013.46)
Supplement: Supplementary Table Legends [file msb201346-s2.docx]

**Table S1**

Variables and parameters of the model.

**Table S2**

Sequences of the PCR primers and oligonucleotide competitors.
